# A vision for an academic health science centre: A survey of research engagement and barriers

**DOI:** 10.1371/journal.pone.0347753

**Published:** 2026-05-08

**Authors:** Gerry Hughes, Claire Temple, Marie E. Ward, Cormac Kennedy, David Kevans, Derval Reidy, Jeremy Towns, Edel O’ Dea, Kishor Santhosh, Catherine Ludden, Mary Day, Martina Hennessy

**Affiliations:** 1 Wellcome-Health Research Board Clinical Research Facility, St James’s Hospital, Dublin, Ireland; 2 School of Medicine, Faculty of Health Sciences, University of Dublin Trinity College, Dublin, Ireland; 3 Research and Innovation Programme, St James’s Hospital, Dublin, Ireland; 4 Quality and Safety Improvement Directorate, St James’s Hospital, Dublin, Ireland; 5 Centre for Innovative Human Systems, School of Psychology, Faculty of Arts, Humanities and Social Sciences, University of Dublin Trinity College, Dublin, Ireland; 6 Corporate Directorate, St James’s Hospital, Dublin, Ireland; Chattogram Veterinary and Animal Sciences University, BANGLADESH

## Abstract

**Background:**

Integrating clinical research within patient care environments has been shown to enhance patient outcomes, foster a culture of clinical inquiry among staff, and improve hospital efficiency.

This study aimed to evaluate the self-reported knowledge, beliefs and attitudes of hospital staff to organisational research and innovation at a large inner-city tertiary referral centre in Dublin, Ireland.

The hospital is undergoing a physical and service expansion on its journey to becoming an academic health science centre.

**Methods:**

A 26-item semi-qualitative survey, iteratively refined by the hospital Research Steering Committee, was disseminated to hospital staff through electronic and face-to-face recruitment, achieving 640 responses. Quantitative data were analysed in R and Microsoft Excel. Content analysis was carried out on qualitative data.

**Results:**

Findings revealed that while most staff recognise the clinical value of research and have engaged in research activities, important barriers exist. Predominant challenges include time constraints, limited research support, and inadequate resources. Despite these barriers, over half of the respondents expressed a strong interest in additional research training, emphasising the need for structured support, including protected time for research, statistical assistance, and enhanced patient engagement initiatives.

**Discussion:**

The results highlight both strengths and limitations within the hospital’s current research culture. While a positive foundation exists, with evident interest in research among staff, infrastructural and logistical gaps must be addressed to facilitate greater engagement. Targeted interventions, such as streamlined research approval processes, resource allocation, and strategic support for multidisciplinary collaboration, could enhance the hospital’s capacity to integrate research into clinical practice. These findings contribute to the ongoing discourse on embedding research in healthcare systems, underscoring the pivotal role of institutional commitment in nurturing a sustainable research culture.

## Introduction

Integrating clinical research to patient care has consistently demonstrated superior outcomes, including lower mortality rates and enhanced patient experiences, compared to care delivered without a research focus [1,2]. In hospital settings, fostering a culture that supports research-active clinicians and involving patients and the public in research enhances clinical activity and performance [2]. Research-active healthcare institutions, particularly those engaged in clinical trials, report lower mortality rates; put simply by Jonker and Fisher: “a correlation between increased research and reduced deaths” [3]. Multiple interdependent drivers, including staffing and resource utilisation efficiencies, likely contribute to this phenomenon [4].

Patients who receive care in research-active hospitals report better communication from clinical staff, more confidence in their clinicians and an overall better patient experience [1,5]. Research co-production, involving stakeholders in collaboration, emerges as another influential process contributing to health outcome improvements in research-active healthcare settings. This collaborative approach bridges gaps between research and care, addressing clinician needs and policymaker goals, ultimately enhancing overall healthcare quality [6]. From a workforce perspective, research-active institutions report reductions in staff turnover, improved staff satisfaction in the workplace as well as an overall improvement in organisational efficiency [7].

Academic health science centres (AHSCs) represent coordinated partnerships between healthcare providers and academic institutions, integrating teaching, training, research, and innovation into clinical practice. This model fosters a dynamic, multidisciplinary workforce and delivers high-quality patient care informed by cutting-edge research [8–10].

AHSCs benefit from a model of knowledge mobilisation, as distinct from knowledge translation, as it aligns closely with their mission to integrate research seamlessly with care delivery [11,12]. Traditional knowledge translation assumes a linear process of applying research findings to practice. In contrast, knowledge mobilisation emphasises the co-creation and real-time integration of knowledge, involving researchers and practitioners in iterative, context-sensitive collaboration. This approach fosters stronger relationships between stakeholders, ensuring that innovations are relevant and readily adopted in clinical care settings.

Embedding such research within complex healthcare organisations requires a robust research culture, defined by shared values and assumptions that prioritise inquiry and innovation [13,14]. Understanding local research awareness and engagement is critical in identifying barriers to participation, aligning institutional strategies with staff needs, and facilitating the cultural shift necessary for AHSC success. A strong research culture enhances the institution’s ability to translate evidence into practice, improving care quality and patient outcomes.

Successful integration of research into clinical environments depends not only on organisational strategies but also on clinicians’ lived experiences within dual clinical–academic roles. Clinical academics may have trouble reconciling clinical service demands with research expectations, challenges in forming a cohesive professional identity, and the need for supportive research cultures and role modelling to enhance wellbeing and engagement. Conducting research may be further hindered by limited research skills, insufficient managerial support, and departmental cultures that do not consistently prioritise research activity [15,16].

It is not only timely, but necessary, to investigate the extant research culture at our institution, with a view to strengthening it in line with the hospital’s overall strategic and clinical research ambition to become an AHSC. The study site is a 1000 bed, urban, adult, acute, publicly funded tertiary referral centre in Dublin, Ireland. The hospital is currently in a state of rapid infrastructural and programmatic expansion with the imminent translocation of paediatric care to the hospital campus, establishment of a comprehensive cancer centre and planning for future incorporation of maternity and infant care. Comparatively, Ireland aligns with wealthier European nations, boasting a relatively high life expectancy and a balanced relationship between economic prosperity (indicated by gross domestic product) and positive health outcomes. While Ireland is regularly benchmarked against the UK, its closest geographical neighbour, persistent differences remain in national healthcare investment, coordination, and cross‑sector collaboration, including healthcare research [17].

## Aim

Evaluate the self-reported knowledge, beliefs and attitudes of hospital staff to organisational research and innovation at a large inner-city tertiary referral centre in Dublin, Ireland.

## Methods

Cross-sectional survey design.

### Development of the survey instrument

A 26-item semi-qualitative survey (S1 Survey Instrument in S1 File) was designed to explore knowledge, beliefs and attitudes of hospital staff towards research and innovation in the workplace. Questions for the survey were developed *de novo* by members of the hospital’s Research Steering Committee. Logic rules allowed participants to bypass questions that did not apply to them. The survey was piloted to assess its usability and face validity on a subset of staff (n = 4) before dissemination. All suggested changes from these reviews were implemented.

### Sampling and recruitment

Convenience sampling was chosen as the preferred sampling method, and the survey was disseminated in two phases. The first survey phase was hosted on Survey Monkey ® and distributed through the hospital’s internal email and intranet system:

- Participants were emailed participant information leaflets and invited to participate using an electronic survey link. All hospital staff were sent two electronically disseminated reminders.
- The survey was also advertised on the usual hospital internal communication channels

To augment the response rate, a second phase recruited participants for completion of a paper-based version of the survey. Departmental managers were provided paper surveys to distribute amongst their staff and a collection box was provided to retrieve completed surveys.

Recruitment was conducted between 01/08/22–30/09/22.

### Data management and analysis

Electronic data was downloaded from Survey Monkey ® to Microsoft Excel ® (ME), hard-copy data was recorded on ME, and the two datasets were merged, inspected and cleaned. Missing data was not imputed. Demographic data were analysed and presented descriptively using R version 4.2.3 (scripts and packages used are detailed in S1 R Script in S2 File) and quantitative survey data were analysed by ME Power Query.

Percentage responses were calculated out of the total number of responses to each question as indicated.

Content analysis was carried out on qualitative data from the open-ended questions by three members of the research team (GH, CT, and MEW). Survey items 10, 12 and the free text option for item 17 were combined and analysed together to provide a deeper insight to barriers to staff engagement with research. Content analysis was deemed the most suitable method for qualitative data analysis, as the survey instrument was not designed to extract latent meaning. [18].

The Checklist for Reporting of Survey Studies (CROSS) quality appraisal tool for carrying out web-based and non-web-based surveys was used (S1 Table CROSS Checklist) [19].

### Ethics and research governance

The hospital’s research and innovation programme (ref 7475) and research ethics committee (REC, ref 1910) approved this study.

Participation was on a voluntary basis. No personal data was collected from participants. As surveys were completed anonymously and voluntarily, participants’ informed consent was implied through the completion of the survey. Therefore, the REC waived the requirement for consent.

All participants in the study were adults and assumed to be in a position to give their own informed consent.

## Results

There were 640 responses (response rate 11%) from an estimated 5604 staff working across the hospital network (Personal Communication, Human Resources April 2023). However, this response rate is likely underestimated as it cannot be confirmed if all staff across the hospital received notification of the survey. Respondent demographics (Table 1) were compared to demographics of the overall hospital staff population (Personal Communication, Human Resources Dept, April 2023) where data was available.

**Table 1:** Demographics.

Staff Category	Survey Respondents (n = 640)	Total Hospital Workforce (n = 5604)
	n (%)	n (%)
Management/Admin/ICT	167 (26.1%)	791 (14.1%)
Nursing	158 (24.7%)	1973 (35.2%)
Allied Health and Social Care	125 (19.5%)	830 (14.8%)
Medical/Dental	101 (15.8%)	690 (12.3%)
General Support	43 (6.7%)	403 (7.2%)
Other	21 (3.3%)	Unknown
Research Staff	18 (2.8%)	Unknown
Patient/Client Care	5 (0.8%)	561 (10.0%)
Unknown	2 (0.3%)	Unknown
**Grade**		
Staff Grade	204 (31.9%)	Data unavailable
Manager	150 (23.4%)	
Senior Practitioner	93 (14.5%)	
Specialist	68 (10.6%)	
Consultant	50 (7.8%)	
NCHD	41 (6.4%)	
Other	19 (3.0%)	
Researcher	9 (1.4%)	
Student	3 (0.5%)	
Unknown	3 (0.5%)	
**Gender**		
Female	474 (74.1%)	4266 (76.1%)
Male	159 (24.8%)	1338 (23.9%)
Prefer not to answer	6 (0.9%)	N/A
Unknown	1 (0.2%)	N/A
**Ethnicity**		
White Irish	540 (84.4%)	3995 (71.3%)
Other White Background	43 (6.7%)	315 (5.6%)
Indian/Pakistani/Bangladeshi	24 (3.8%)	667 (11.9%)
Other Asian Background	15 (2.3%)	408 (7.3%)
Black African	9 (1.4%)	170 (3%)
Other	4 (0.6%)	7 (0.1%)
Arabic	3 (0.5%)	26 (0.5%)
Chinese	1 (0.2%)	16 (0.3%)
Unknown	1 (0.2%)	N/A
**Time working in the hospital**		
>15 years	259 (40.5%)	1560 (27.8%)
<1 year	82 (12.8%)	1070 (19.1%)
3-5 years	80 (12.5%)	459 (8.2%)
1-3 years	79 (12.3%)	1485 (26.5%)
10-15 years	58 (9.1%)	330 (5.9%)
5-7 years	49 (7.7%)	418 (7.5%)
7-10 years	31 (4.9%)	281 (5.0%)
Unknown	2 (0.3%)	1 (0.0%)
**Highest Level of Education Achieved**		
Postgraduate Diploma or Master’s Degree	261 (40.8%)	Data unavailable
Honours Bachelor Degree/Professional Qualification or Both	126 (19.7%)	
Doctorate (MD or PhD or higher)	78 (12.2%)	
Upper Secondary	52 (8.1%)	
Ordinary Bachelor Degree or National Diploma	42 (6.6%)	
Higher Certificate	30 (4.7%)	
Technical or Vocational	19 (3.0%)	
Advanced Certificate/ Completed Apprenticeship	14 (2.2%)	
Lower Secondary	12 (1.9%)	
Primary education	2 (0.3%)	
No formal education/training	1 (0.2%)	
Unknown	3 (0.5%)	Data unavailable

**ICT**: information and communication technology; **MD**: Doctor of Medicine; **NCHD**: non-consultant hospital doctor, doctor in training; **PhD**: Doctor of Philosophy.

### Experience of research

**Fig 1:**
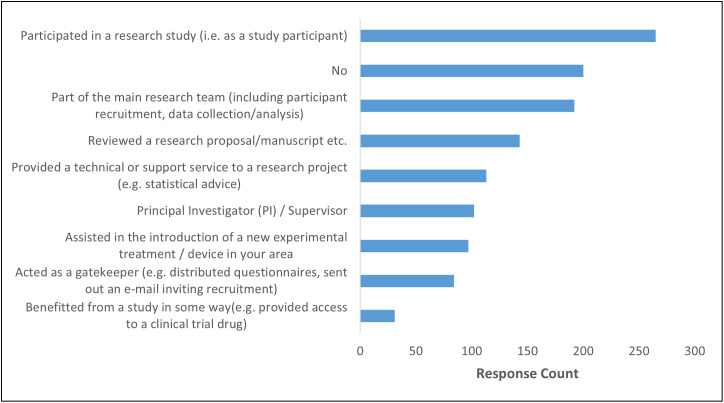
Participants’ experience of being involved with research.

Most respondents had engaged with research in some way (Fig 1). For this question, respondents were free to choose more than one response.

### Engagement with research

**Fig 2:**
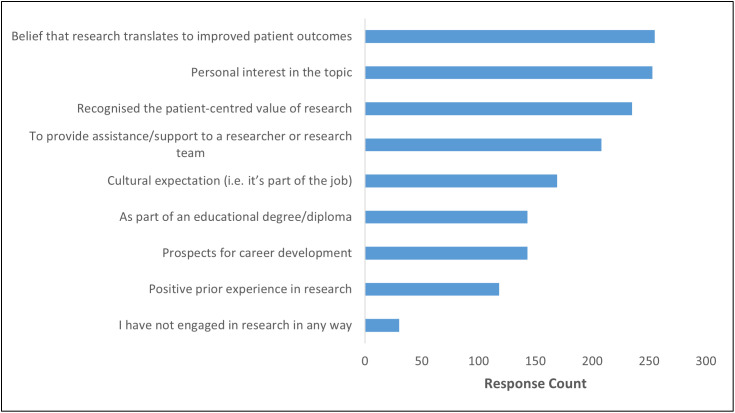
Participants’ engagement with research activity.

Participants mostly became involved with research in recognising potential for translation to clinical practice, for their own personal interest or because they saw the patient-centred value (Fig 2). Most participants were also interested in becoming involved with research at some point in the future (S1 Fig), while over half of respondents had not completed any formal research training (S2 Fig). Participants were asked to give further details on the types of training they received (S2 Table).

Barriers to engagement with research (Table 2) mostly related to lack of protected time, resources, research support ora general lack of interest in conducting research.

**Table 2:** Barriers to research engagement*.

Barrier Identified	Responses (n) out of total of 20 identified barriers	Proportion (%) of total responses to this question
Time constraints	47	30
Lack of research support (including from management)	18	12
Not interested currently	13	8
Not enough resources	12	8
Institutional and ethical approval processes	12	8

*: Top 5 identified barriers, full table available in S3 Table

### Perception of a research active hospital

**Fig 3:**
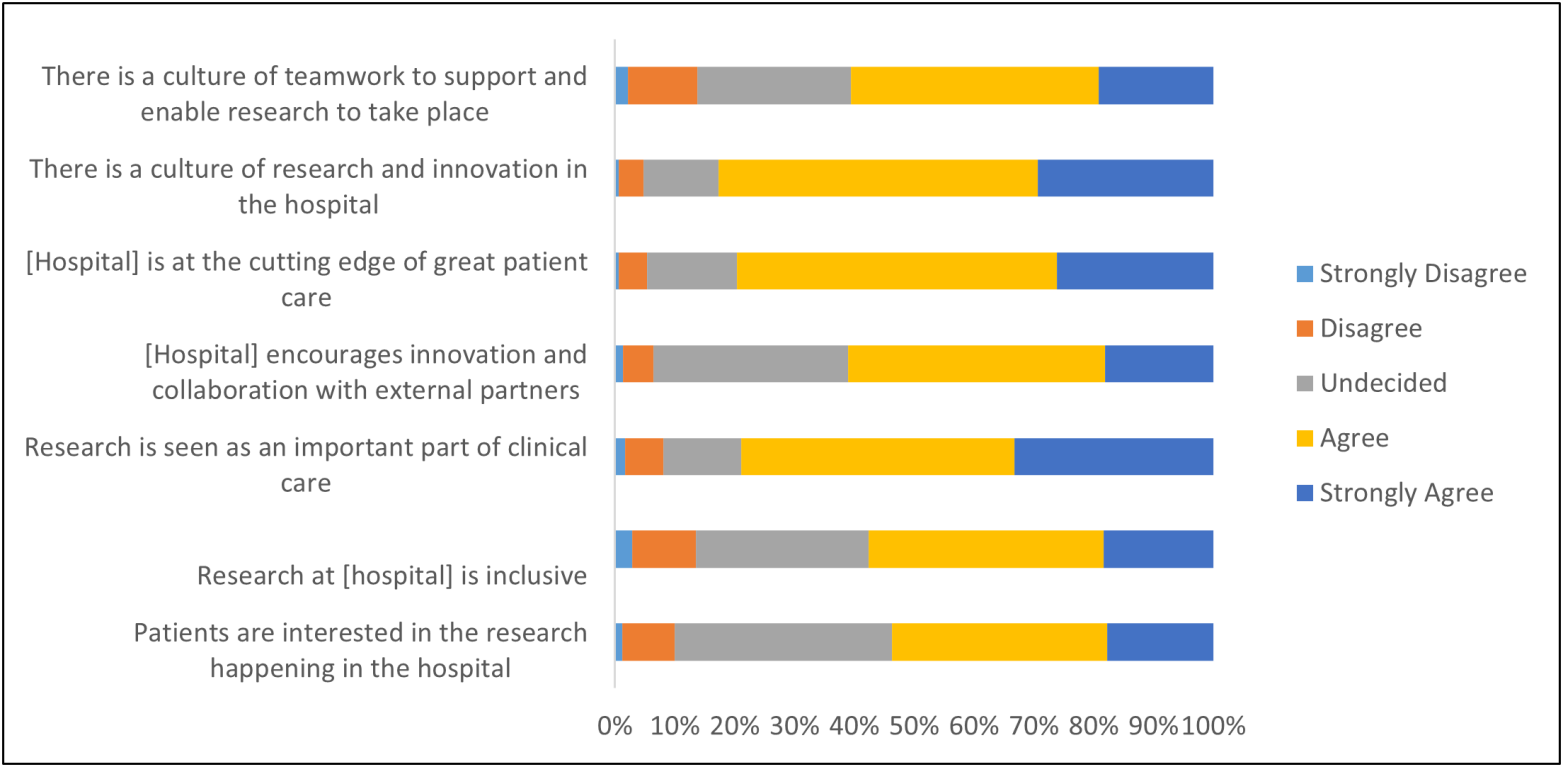
Pride in research-related activity.

In considering research activity, participants rated statements relating to pride in the workplace. Most respondents recognised a positive research culture, and that the hospital is at the cutting edge of research while approximately half of respondents agreed that the hospital encourages innovation and collaboration and inclusivity. However, only around 50% of respondents thought that patients are interested in research activity occurring at our institution (Fig 3).

**Fig 4:**
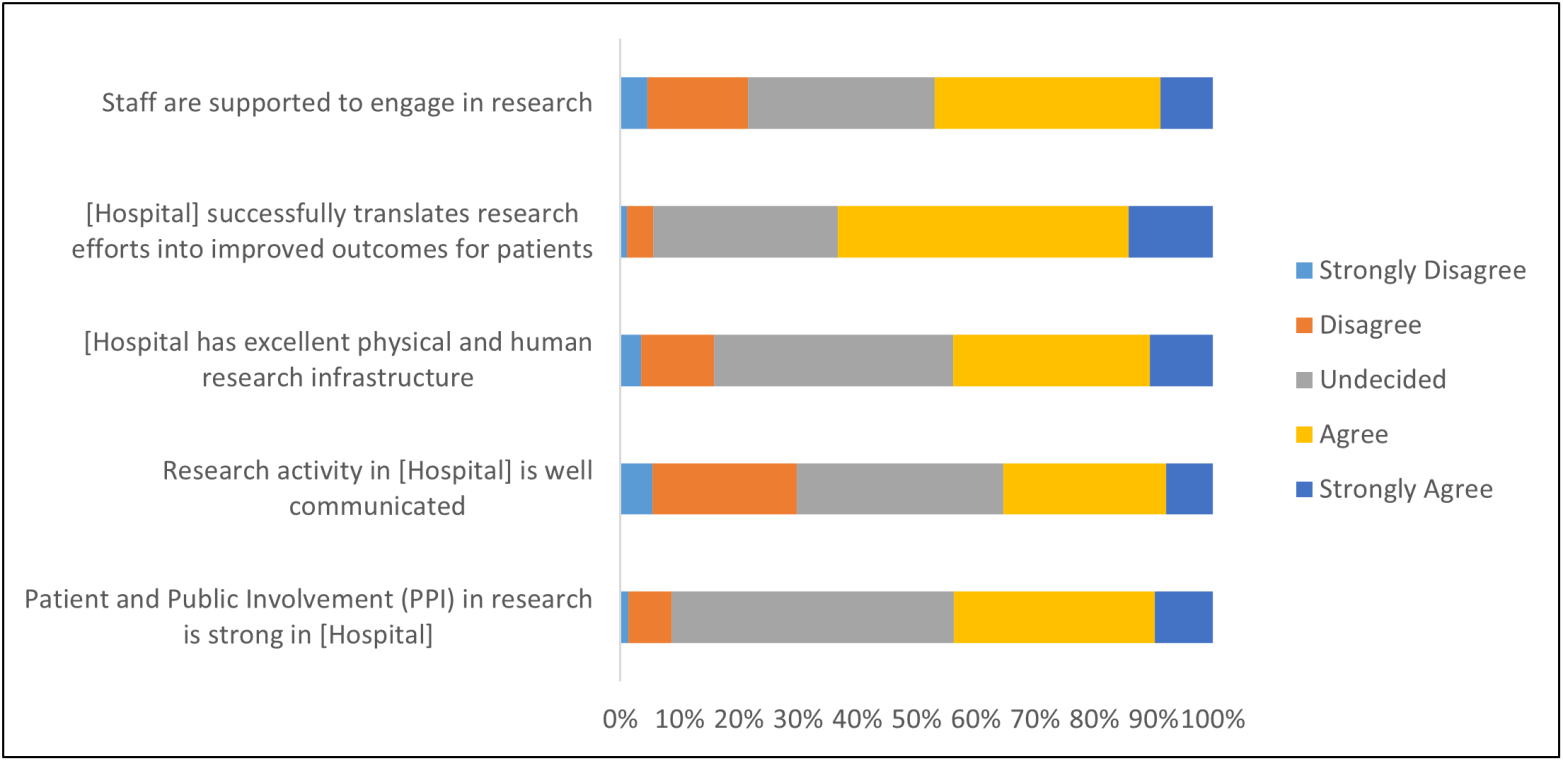
Perceptions of how the hospital ‘is doing’ related to research.

Approximately 45% of respondents reported that hospital staff are sufficiently supported to engage in research and that there is commensurate hospital infrastructure to do so. Communicating research activity and involving patients and the public in research were also identified as areas for improvement (Fig 4).

Participants were asked to elaborate on any basic infrastructure or supports needed. There were 67 responses coded into 12 categories (Table 3).

**Table 3:** Infrastructure and supports needed.

Category	Illustrative Quotation
Statistics	*“…statistical support including software and expertise.”*
Research support staff general	*“Designated support persons who are highly skilled in conducting research that can act as liaison persons for staff in areas who are not as skilled/knowledgeable to provide assistance and guidance. Similar to a supervisor for a thesis.”*
Data and IT	*“Bioinformatics and biostatistics needed across campus. Data experts, integration and support for ICT in this area.”*
More general staff (to free up time)/Protected time for research	*“Protected time for research is needed to allow nursing be more involved in research.”* *“it is necessary for the hospital to also have their own similar dedicated research staff to support research activities (e.g., science communications officer, statistician, PPI advocate, nursing research lead, funding for automated biobank systems). This will be looked at more favourably by the clinical team and they will see how research is embedded.”*
Research support staff nursing/AHP	*“Dedicated support for research is also needed for Allied Health Care staff (Dietitians, social workers, Clinical Physiologists, Physiotherapists, etc.)*
Ethics Support	*“Fast track ethics. Assistance in filling out forms and designing robust projects.”*
Training	*“Training and time for all staff if interested to engage in research.”*
Access to library	*“Access to scientific publications is another big problem.*
Consumables/Scans	*“Basic funding for consumables must be provided for staff engaging in translational research.”*
Communication	*“Knowledge bank via intranet or weekly newsletter articles encouraging participation / research projects taking place.”*
Streamlined consent processes	*“Support to systematically/automatically request patient consent would be helpful.*
Space for research	*“Administrative support and more space.”*

Respondents provided their insights into the hospital’s strategic research priorities (S3–S7 Figs) and how the hospital could achieve these ambitions (S4 Table). Consistent with overarching findings, these responses shed light on facets of the research culture and infrastructure at our institution warranting improvement. Participants responded positively to integration of research with clinical care, emphasising a multi-disciplinary approach for optimal support. While respondents advocated for patient and public partnership (PPP) in research, they underscored an existing knowledge and information gap hindering effective PPP. Recognition of the imperative for clinical academic posts was evident; however, respondents highlighted a lack of institutional support for such positions.

Training and educational considerations featured prominently, with participants expressing a collective demand for statistical support for research studies. The proposed availability of electronic educational and training content garnered favourable responses.

## Discussion

This study yields valuable insights into the organisational research culture at our institution, highlighting that most respondents have engaged in research, motivated by translational potential, personal interest, and perceived patient-centred value. These findings provide a first-time comprehensive assessment of the hospital’s research landscape. Specific areas requiring attention and refinement are also revealed within the context of strategic research priorities; previous research highlights the importance of addressing these issues towards achieving a healthy organisational research culture [2,7]. These include time constraints, lack of research support, and resource limitations; results which also echo recognised challenges reported internationally [13]. As the survey also captured staff across non‑traditional clinical research roles, it provides an alternative perspective on organisational research culture. The findings illustrate how research is perceived and experienced at the frontline of patient care, highlighting gaps between staff interest in research and the structural supports required to enact it. Staff see research as an important contributor to high‑quality clinical care, consistent with the emerging ‘research as care’ paradigm. However, the persistent structural barriers identified indicate that research is not yet embedded in routine clinical workflows.

Building upon our findings, the barriers identified are consistent with challenges reported in the broader literature. A scoping review by Yoong *et al* highlights that healthcare providers frequently encounter systemic obstacles that hinder their engagement in research, such as insufficient institutional support and the competing demands of clinical duties. These barriers not only impede research participation but also contribute to clinician frustration and disengagement from academic activities [20]. Our findings also resonate with Boucher *et al*, who identified that frontline clinicians often feel disconnected from research activities, largely due to these pervasive challenges. This disconnect underscores the importance of fostering an inclusive research culture that actively engages clinicians and addresses structural barriers [21]. Mickan expands on this study by highlighting how prioritising research through leadership action and clinician-focused strategies can yield tangible benefits, including improved staff retention and enhanced patient care [22].

Our results sit within a rapidly evolving academic landscape for the hospital, shaped in part by its close relationship with the hospital’s academic partner Trinity College Dublin. That wider ecosystem is marked by considerable research activity: more than 800 investigators, 163 PhD students, 21 scientific disciplines, over 6,000 publications between 2015 and 2020, and roughly €20 million in clinical research income [23]. Against this backdrop, our results reflect the lived experiences of staff working within a system that is increasingly orienting toward an AHSC model, highlighting the need to build a workforce that is supported, prepared, and able to engage meaningfully with this expanding research environment

Findings from our study should also be considered in context with the wider clinical research ecology in Ireland. Although this study did not examine staff perceptions or experiences with GDPR or the Health Research Regulations, these frameworks shape the wider context in which research activity occurs in Ireland [24–26]. Respondents in our survey frequently reported administrative burden, delays, and time constraints as barriers to engaging in research. National discourse has highlighted that GDPR‑related procedural complexity can contribute to these types of organisational bottlenecks, suggesting that some of the difficulties identified by staff may be indirectly influenced by the regulatory landscape rather than local processes alone [27]. For this reason, our findings are best understood as situated within both institutional and national structural conditions that collectively affect research participation.

A 2022 joint position statement by the Royal College of Physicians and the National Institute for Health and Care Research argued for “making research everybody’s business” and proposed key recommendations to achieve that [28]. Arguably the most notable recommendation is a shift in clinical environment culture where clinical and corporate actors are more aligned towards the facilitation and support of research. Findings from our work have since guided research leaders at our institution in designing and implementing interventions which aim to address the fragmented research administration which participants alluded to in their responses.

The hospital has recently implemented several initiatives to strengthen research infrastructure, including an integrated electronic ethics and data protection oversight system that streamlines applications and reduces administrative burden, and the adoption of national model clinical trial agreements to improve trial start‑up efficiency [29]. The Clinical Research Facility has enhanced its capacity through the establishment of a scientific advisory board, the creation of a clinical research statistics role, and expanded partnerships (including industry collaborations) focused on value‑based healthcare and AI‑driven data analysis. In addition, new joint university/hospital training opportunities now provides practical education in research methods, grant development, academic writing, and patient and public partnership.

Our findings have relevance not only for our institution but also for hospitals operating in similar large, complex healthcare environments. Many of the barriers identified are common across acute hospitals seeking to strengthen research culture [30]. To support a more research‑active organisational environment, hospitals in comparable settings may consider the following actions:

1. **Allocating protected time for research:** While most healthcare professional employment contracts encourage participation in research activity it is not currently mandated.
2. **Providing research support:** This can include training programs to enhance research skills, further providing resources, and fostering a supportive management culture
3. **Streamlining approval processes:** Making institutional and ethical approval processes more efficient can encourage more healthcare professionals to engage in research
4. **Promoting the benefits of research**: Highlighting the positive impact of research on patient outcomes and professional development can increase interest in research
5. **Encouraging collaboration:** Fostering a culture of collaboration and communication can make research activities more efficient and effective
6. **Keeping up-to-date with legislation**: Regularly updating staff on changes in legislation can help them navigate any legal requirements for conducting research

These recommendations mirror several national priorities identified by the recently published Irish Department of Health National Clinical Trials Oversight Group report [31].

### Strengths and limitations

Our study offers a robust examination of the organisational research culture at the hospital, providing a comprehensive overview of staff knowledge, beliefs, and attitudes. The response rate, albeit conservative due to survey dissemination uncertainty, contributes to the institutional understanding of how research is aligned with clinical care. A multi-faceted approach, incorporating electronic and face-to-face survey dissemination, enhances inclusivity across diverse staff categories. Qualitative enquiry enriches the quantitative findings, presenting a nuanced understanding of barriers and aspirations.

Although the overall sample size was substantial, some staff groups were under‑ or over‑represented relative to the hospital workforce, which may influence the generalisability of findings across professional categories.

The likely underestimated response rate of 11% may introduce a selection bias, further potentially influencing the generalisability of findings. However, because the survey distribution pathways varied, the true denominator of staff reached is uncertain, which limits the precision of the response rate estimate. The survey relied exclusively on self‑reported perceptions without triangulation against organisational data or operational performance metrics. Perspectives on research are likely to differ meaningfully between the heterogeneous group of participants in this study; therefore, the results should be interpreted as reflecting broad institutional sentiment rather than the views of research‑active professionals specifically. Cross-sectional survey designs limit causal inferences, capturing a snapshot of data at a specific point. Finally, the use of convenience sampling may introduce variability, affecting the study’s external validity.

Despite these limitations, the study provides a new and valuable overview of the institutional research landscape.

## Conclusion

Our work provides a valuable insight into the organisational research culture, elucidating both strengths and areas for improvement. The positive alignment of staff motivations with previous research underscores the potential for enhancing clinical outcomes through integrated research practices. Identified barriers, including time constraints and resource limitations, necessitate targeted interventions for a more conducive research environment. Our findings advocate for a strategic shift towards supporting research through protected time, enhanced resources, and institution-wide practical leadership in conducting clinical research. The findings also contribute to the ongoing discourse on aligning evidence-based practice with robust clinical research, emphasising the need for a harmonious ecosystem within healthcare organisations. As the hospital evolves into an AHSC, addressing these findings becomes paramount for achieving a synergistic balance between clinical care and research activities.

## Supporting information

S1 Fig Interest in future research involvement. (DOCX)

S1 File Survey instrument. (PDF)

S2 File R Script. (DOCX)

S1 Table CROSS checklist. (DOCX)

S2 Fig Completed research training. (DOCX)

S2 Table Research training undertaken by participants. (DOCX)

S3 Fig Participant feedback on research priority 1. (DOCX)

S3 Table Barriers to research engagement. (DOCX)

S4 Fig Participant feedback on research priority 2. (DOCX)

S4 Table Participant suggestions for strategic research priorities. (DOCX)

S5 Fig Participant feedback on research priority 3. (DOCX)

S6 Fig Participant feedback on research priority 4. (DOCX)

S7 Fig Participant feedback on research priority 5. (DOCX)
