# Supplementary figures and images for: A vision for an academic health science centre: A survey of research engagement and barriers
Source: PLoS One. 2026 May 8;21(5):e0347753. doi: 10.1371/journal.pone.0347753 (PMC13155618; doi:10.1371/journal.pone.0347753)

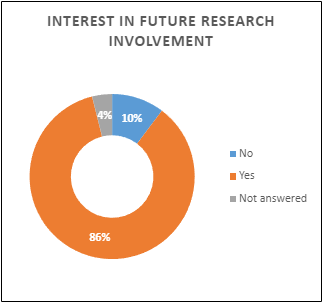


**S1 Figure:** Interest in future research involvement

Supplement: S1 Fig — (DOCX) [file pone.0347753.s001.docx]
