# Supplementary material for: A vision for an academic health science centre: A survey of research engagement and barriers
Source: PLoS One. 2026 May 8;21(5):e0347753. doi: 10.1371/journal.pone.0347753 (PMC13155618; doi:10.1371/journal.pone.0347753)
Supplement: S1 File — (PDF) [file pone.0347753.s002.pdf]

Staff survey to inform the St James's Hospital Research Strategy

Section 1: Introduction

Development of a Research Strategy for St James's Hospital

St James's Hospital Dublin has a strong commitment and ambition to deliver research that has the potential to benefit our patients and society. The Hospital is expanding and changing, and we would like to know what you think the future of research will look like and how it will fit with clinical care.

We are currently developing a strategy to shape the future of research over the coming years and would like your input and feedback. This survey has been designed to understand the research culture in St James's and the priorities that are important for our staff and patients.

The survey should take approximately 12 minutes to complete, all responses will be completely anonymous.

Please contact research@stjames.ie if you have any queries.

Staff survey to inform the St James's Hospital Research Strategy

Section 2: 'All About You'

* 1. **Which staff category best describes your role?**

◯ Medical/Dental

◯ Nursing

◯ Management/Admin/Information Communication Technology

◯ Allied Health and Social Care

◯ General Support

◯ Patient/Client Care

◯ Research Staff

◯ Other (please specify)

* 2. **Are you:**

◯ Directly employed by St James's Hospital

◯ External staff member (e.g. agency staff)

◯ Other (please specify)

* 3. **What grade/level best describes your role?**

◯ Staff Grade

◯ Senior

◯ Manager

◯ Specialist

◯ Non Consultant Hospital Doctor (NCHD)

◯ Consultant

◯ Student

◯ Researcher

◯ Other (please specify)

* 4. **With which gender do you identify?**

◯ Male

◯ Female

◯ Prefer not to answer

* 5. **What is your ethnic group/ background?**

◯ White Irish

◯ Irish Traveller

◯ Roma

◯ Other White Background

◯ Black African

◯ Other Black Background

◯ Chinese

◯ Indian/Pakistani/Bangladeshi

◯ Other Asian Background

◯ Arabic

◯ Other: Please specify

* 6. **How long have you worked in St James's Hospital?**

- ◯ <1 year
- ◯ 1-3 years
- ◯ 3-5 years
- ◯ 5-7 years
- ◯ 7-10 years
- ◯ 10-15 years
- ◯ >15 years

* 7. **What is your HIGHEST level of education?**

- ◯ Doctorate (MD or PhD or higher, NFQ Level 10)
- ◯ Postgraduate Diploma or Masters Degree (NFQ Level 9)
- ◯ Honours Bachelor Degree/Professional Qualification or Both (NFQ Level 8)
- ◯ Ordinary Bachelor Degree or National Diploma (NFQ Level 7)
- ◯ Higher Certificate (NFQ Level 6)
- ◯ Advanced Certificate/ Completed Apprenticeship (NFQ Level 6)
- ◯ Technical or Vocational (NFQ Levels 4 or 5)
- ◯ Upper Secondary (NFQ Levels 4 or 5, Leaving Certificate)
- ◯ Lower Secondary (NFQ Level 3, Junior/Inter/Group Certificate)
- ◯ Primary education (NFQ Levels 1 or 2)
- ◯ No formal education/training

* 8. **Have you any experience of working/participating in/benefiting from research projects at SJH?**

This can include being involved in any aspect of a research project– it is not limited to those on the main research project team.

Please select all that apply:

- [ ] Participated in a research study (i.e. as a study participant)
- [ ] Benefitted from a study in some way(e.g. provided access to a clinical trial drug)
- [ ] Assisted in the introduction of a new experimental treatment / device in your area
- [ ] Principal Investigator (PI) / Supervisor
- [ ] Part of the main research team (including participant recruitment, data collection/analysis)
- [ ] Acted as a gatekeeper (e.g. distributed questionnaires, sent out an e-mail inviting recruitment)
- [ ] Reviewed a research proposal/manuscript etc.
- [ ] Provided a technical or support service to a research project (e.g. statistical advice)
- [ ] No
- [ ] Other (please specify)

Staff survey to inform the St James's Hospital Research Strategy

* 9. **Why did you decide to engage in research?**
Please select all that apply:

- [ ] Cultural expectation (i.e. it's part of the job)
- [ ] Personal interest in the topic
- [ ] Recognised the patient-centred value of research
- [ ] Prospects for career development
- [ ] As part of an educational degree/diploma
- [ ] Belief that research translates to improved patient outcomes
- [ ] Positive prior experience in research
- [ ] To provide assistance/support to a researcher or research team
- [ ] I have not engaged in research in any way
- [ ] Other (please specify)

Staff survey to inform the St James's Hospital Research Strategy

* 10. **If you have never engaged in any research activity, including supporting research, what has been the main barrier?**

Staff survey to inform the St James's Hospital Research Strategy

* 11. **Would you be interested in getting involved in research projects in the future?**

◯ Yes

◯ No

Staff survey to inform the St James's Hospital Research Strategy

* 12. **Why would you not be interested in getting involved in research projects in the future?**

Staff survey to inform the St James's Hospital Research Strategy

* 13. **Have you completed training/education relating to research?**

Examples: GCP training, data protection training for research, survey design, focus group moderation

◯ Yes

◯ No

If yes, please give further details

Staff survey to inform the St James's Hospital Research Strategy

* 14. **If not, would you be interested in accessing training/education related to research in the future which is appropriate to your role?**

◯ Yes

◯ No

Staff survey to inform the St James's Hospital Research Strategy

Section 3: 'All About the Culture'

Exploring the Culture of Research at St James's Hospital

* 15. **Regarding research, which of the following makes you proud to work in St James's Hospital?**
Please rate your level of agreement with each of the following:

	Strongly Agree	Agree	Undecided	Disagree	Strongly Disagree
There is a culture of research and innovation in the hospital	◯	◯	◯	◯	◯
St James's Hospital is at the cutting edge of great patient care	◯	◯	◯	◯	◯
Research at St James's Hospital is inclusive	◯	◯	◯	◯	◯
St James's Hospital encourages innovation and collaboration with external partners	◯	◯	◯	◯	◯
There is a culture of teamwork to support and enable research to take place	◯	◯	◯	◯	◯
Research is seen as an important part of clinical care	◯	◯	◯	◯	◯
Patients are interested in the research happening in the hospital	◯	◯	◯	◯	◯

* 16. **Are you aware of any research that has taken place in your department/area of work?**

◯ Yes

◯ No

If yes, please give further details

* 17. **What would you see as the barriers most likely to impact you engaging in research activity in your current role?**
Please rate your level of agreement with each of the following:

	Very Likely	Somewhat Likely	No Impact	Unlikely	Very Unlikely
Busy workload	◯	◯	◯	◯	◯
Don't know where to start	◯	◯	◯	◯	◯
Difficulty in getting funding	◯	◯	◯	◯	◯
Not enough training available	◯	◯	◯	◯	◯
Lack of management support	◯	◯	◯	◯	◯
Not enough research support in SJH	◯	◯	◯	◯	◯
My lack of awareness of research going on	◯	◯	◯	◯	◯

Other (please specify)

* 18. **How are we doing?**

Please rate your level of agreement with each of the following:

	Strongly Agree	Agree	Undecided	Disagree	Stronly Disagree
SJH successfully translates research efforts into improved outcomes for patients	◯	◯	◯	◯	◯
Patient and Public Involvement (PPI) in research is strong in SJH	◯	◯	◯	◯	◯
Staff are supported to engage in research	◯	◯	◯	◯	◯
SJH has excellent physical and human research infrastructure	◯	◯	◯	◯	◯
Research activity in SJH is well communicated	◯	◯	◯	◯	◯

Staff survey to inform the St James's Hospital Research Strategy

This final section relates to the 5 Strategic Priorities of the Research Strategy

* 19. **Strategic priorities for research**

The strategic priorities will be the key focus areas over the coming years.

From your perspective, please rank the priorities below in terms of their importance from 1-5.
Please rank these in order of priority: 1 being the most important, 2 the next most important etc.

- Translating our research efforts into improved outcomes for patients.
- Encouraging both patient participation and patient involvement in research.
- Supporting our staff to engage in research.
- Building on and improving the hospital's basic research infrastructure (This includes biobanking, patient registries and other research supports).
- Effectively communicating our research.

20. **Please outline any other areas that you think should be designated as a strategic priority:**

* 21. **<u>Priority 1</u>: We aim to focus on translating our research efforts into improved outcomes for patients.**
Please rate your level of agreement with each of the following:

	I am not informed enough about this topic to provide an answer	Strongly Agree	Agree	Undecided	Disagree	Strongly Disagree
Research conducted by SJH improves patients' lives.	○	○	○	○	○	○
Pairing scientific researchers with clinicians would be beneficial for patients.	○	○	○	○	○	○
Research should be integrated more into clinical care.	○	○	○	○	○	○
Protected time is currently available for research.	○	○	○	○	○	○
All disciplines should be encouraged and supported to engage in research.	○	○	○	○	○	○
SJH should champion impactful research in focused disease areas and themes	○	○	○	○	○	○

* 22. **Priority 2: We will encourage both patient participation and patient involvement in research.**

Please rate your level of agreement with each of the following:

	I am not informed enough about this topic to provide an answer	Strongly Agree	Agree	Undecided	Disagree	Strongly Disagree
There is sufficient information available for patients to encourage their participation in research.	○	○	○	○	○	○
A Public and Patient Involvement lead for research would be a useful initiative.	○	○	○	○	○	○
Healthy people should be supported to take part in research.	○	○	○	○	○	○
It would be helpful to have patient advocates for research.	○	○	○	○	○	○

* 23. **<u>Priority 3</u>: We will support, empower and enable our staff to engage in research.**

Please rate your level of agreement with each of the following:

	I am not informed enough about this topic to provide an answer	Strongly Agree	Agree	Undecided	Disagree	Strongly Disagree
The resources and infrastructure available to support research are widely known across the hospital.	◯	◯	◯	◯	◯	◯
There are promotional opportunities available for staff who have undertaken further academic qualifications.	◯	◯	◯	◯	◯	◯
Collaboration is encouraged, with expertise and resources shared, and staff working together to amplify the impact of the research.	◯	◯	◯	◯	◯	◯
There should be more clinical academic posts available in all disciplines.	◯	◯	◯	◯	◯	◯

* 24. **Priority 4: We will build on and improve the hospital's basic research infrastructure**

Please rate your level of agreement with each of the following:

	I am not informed enough about this topic to provide an answer	Strongly Agree	Agree	Undecided	Disagree	Strongly Disagree
Existing structures are utilised fully e.g. biobanks, registries, health systems research support.	◯	◯	◯	◯	◯	◯
Dedicated nursing support for research is needed.	◯	◯	◯	◯	◯	◯
Statistical support for research is needed.	◯	◯	◯	◯	◯	◯

Please elaborate on any other basic infrastructure/supports you think are needed

* 25. **Priority 5: We will effectively communicate our research**

Please rate your level of agreement with each of the following:

	I am not informed enough about this topic to provide an answer	Strongly Agree	Agree	Undecided	Disagree	Strongly Disagree
We should list publications and grants on the hospital website.	◯	◯	◯	◯	◯	◯
Webinars/short videos can be a useful method for communication of research outputs.	◯	◯	◯	◯	◯	◯
SJH staff are suitably acknowledged in publications on externally led studies.	◯	◯	◯	◯	◯	◯

26. **Final thoughts**

In thinking about a research strategy for SJH do you have any other thoughts or comments?

Staff survey to inform the St James's Hospital Research Strategy

Close & Thanks

Thank you for completing this survey. We will be communicating the results through the staff intranet.

Please contact research@stjames.ie if you have any queries.
