# Supplementary material for: A vision for an academic health science centre: A survey of research engagement and barriers
Source: PLoS One. 2026 May 8;21(5):e0347753. doi: 10.1371/journal.pone.0347753 (PMC13155618; doi:10.1371/journal.pone.0347753)
Supplement: S2 File — (DOCX) [file pone.0347753.s003.docx]

**S1: R packages and analysis script**

#Libraries

library(tidyverse)

library(readxl)

library(jtools)

library(gtsummary)

library(openxlsx)

library(scales)

library(knitr)

library(ggrepel)

library(kableExtra)

#Import

df <- read_excel("G:/R&I/Research Strategy/Survey/Analysis/Survey Responses and Analysis/Data/research_strategy_survey_analysisx_inc_freetext.xlsx", col_names = FALSE)

View(df)

#Skip junk rows and rename headings

df_new_heads<- read_excel("G:/R&I/Research Strategy/Survey/Analysis/Survey Responses and Analysis/Data/research_strategy_survey_analysisx.xlsx",

skip = 4, col_names = FALSE)

names(df_new_heads) <- paste0("Q", 1:88)

View(df_new_heads)

#Demographics Summary

demographics<- df_new_heads %>% select(2, 4, 6, 8, 9, 11, 12)

demographics %>%

tbl_summary(label = list("Q2" ~ "Staff Category",

"Q4" ~ "Employee Type",

"Q6" ~ "Grade",

"Q8" ~ "Gender",

"Q9" ~ "Ethnicity",

"Q11" ~ "Time in SJH",

"Q12" ~ "Education"),

sort = list(everything() ~ "frequency")) %>%

as_gt %>%

gt::tab_options(table.font.size = "90%")
