# Supplementary material for: A vision for an academic health science centre: A survey of research engagement and barriers
Source: PLoS One. 2026 May 8;21(5):e0347753. doi: 10.1371/journal.pone.0347753 (PMC13155618; doi:10.1371/journal.pone.0347753)
Supplement: S1 Table — (DOCX) [file pone.0347753.s004.docx]

**S1 Table:** CROSS Checklist

| **Section/Topic** | **Item** | **Item Description** | **Reported in Paper** | **Page Number** | **Explanatory Notes** |
| --- | --- | --- | --- | --- | --- |
| **Title and abstract** | 1a | State the word "survey" in the title/abstract. | Yes | Page 1 | The word "survey" is included in the title and abstract. |
|  | 1b | Provide an informative summary in the abstract. | Yes | Pages 2-3 | Abstract includes background, aims, methods, results, and conclusions. |
| **Introduction** | 2 | Background about the rationale of the study. | Yes | Pages 3-6 | Rationale is provided, highlighting gaps in knowledge of research culture at the hospital. |
|  | 3 | Identify specific aims/objectives. | Yes | Page 6 | Aim to evaluate staff knowledge, beliefs, and attitudes towards research and innovation is stated. |
| **Methods** | 4 | Specify the study design. | Yes | Page 6 | The design is specified as cross-sectional, semi-qualitative survey. |
|  | 5a | Describe the questionnaire (sections/questions). | Yes | Page 6 | The 26-item survey is described, including the use of logic rules for bypassing irrelevant questions; survey instrument provided in supplementary materials. |
|  | 5b | Describe questionnaire instruments used. | Yes | Page 6 | Survey instrument provided in Supplementary Material |
|  | 5c | Provide info on pretesting of the questionnaire. | Yes | Page 6 | Pretesting was conducted with four staff members, and revisions were implemented based on feedback. |
|  | 5d | Include full questionnaire if possible. | Yes | NA | Survey instrument provided in Supplementary Material |
|  | 6a | Describe the study population. | Yes | Page 9 | Study population described as hospital staff across various roles. |
|  | 6b | Describe the sampling techniques. | Yes | Pages 6-7 | Convenience sampling method is described. |
|  | 6c | Provide sample size and calculation details. | Yes | Page 8 | Sample size (n=640) is reported; convenience sampling strategy. |
|  | 6d | How representative is the sample? | Yes | Pages 9 & 24 | Demographics of respondents are compared to overall hospital workforce; also discussed in Limitations. |
|  | 7a | Modes of questionnaire administration. | Yes | Pages 5 & 6 | Administered electronically and via paper-based surveys. |
|  | 7b | Provide survey time frame. | Yes | Page 7 | Conducted in summer 2022. |
|  | 7c | Describe data entry process. | Yes | Page 7 | Electronic data downloaded; hard-copy data manually entered and merged. |
|  | 8 | Preparation process before conducting the survey. | Yes | Pages 6-7 | Includes questionnaire piloting and revisions. |
| **Ethical considerations** | 9a | Provide ethical approval information. | Yes | Page 8 | Ethical approval from the hospital’s research ethics committee and institutional review board is documented. |
|  | 9b | Describe survey anonymity/confidentiality. | Yes | Page 8 | Survey anonymity ensured, and no personal data collected. |
| **Statistical analysis** | 10a | Describe statistical methods used. | Yes | Pages 7 & 8 | Descriptive statistics performed in R and Excel; content analysis for qualitative data. |
|  | 10b | Report any modification of variables. | Yes | Page 7 | Combination of survey items for analysis. |
|  | 10c | Describe handling of missing data. | Yes | Page 7 | Missing data was not imputed, noted in the methodology. |
|  | 10d | Address non-response error. | No | Page 24 | Not calculated |
|  | 10e | Address loss to follow-up (longitudinal). | No | NA | Not applicable for this cross-sectional survey. |
|  | 10f | Adjustments for non-representativeness. | No | Pages 9, 24 & 25 | Adjustments not calculated but referenced in the demographics table and Limitations |
|  | 10g | Describe sensitivity analysis. | No | NA | Sensitivity analysis not performed. |
| **Results** | 11a | Report respondent numbers at each stage. | Yes | Page 8 | Respondent numbers (n=640) and response rate (11%) are reported. |
|  | 11b | Provide reasons for non-participation. | No | NA | Reasons for non-participation are not discussed. |
|  | 11c | Report response rate and calculation formula. | Yes | Page 8 | Response rate calculation is included. |
|  | 11d | Define unique visitors (if relevant). | No | NA | Not applicable for this study. |
|  | 12 | Characteristics of study participants. | Yes | Page 9 | Participant demographics are detailed in Table 1. |
|  | 13a | Provide unadjusted estimates (if applicable). | No |  | Not applicable. |
|  | 13b | Multivariable analysis details. | No |  | Not applicable. |
|  | 13c | Details of sensitivity analysis. | No |  | Sensitivity analysis not included. |
| **Discussion** | 14 | Discuss study limitations. | Yes | Page 24-25 | Limitations include low response rate, self-reported data, and sampling bias. |
|  | 15 | Provide cautious overall interpretation. | Yes | Pages 24-25 | Interpretation considers limitations and contextualizes findings. |
|  | 16 | Discuss external validity. | Yes | Page 22-23 | External validity discussed in terms of generalisability. |
| **Other sections** | 17 | State funding organisation roles. | NA |  |  |
|  | 18 | Declare conflicts of interest. | Yes |  | Conflicts of interest addressed as part of manuscript submission. |
|  | 19 | Acknowledge contributors. | Yes | Pages 1 & 32 | Author contributions addressed as part of manuscript submission |
