# Supplementary material for: A vision for an academic health science centre: A survey of research engagement and barriers
Source: PLoS One. 2026 May 8;21(5):e0347753. doi: 10.1371/journal.pone.0347753 (PMC13155618; doi:10.1371/journal.pone.0347753)
Supplement: S2 Table — (DOCX) [file pone.0347753.s006.docx]

S2 Table: Research training undertaken by participants

| **Training Type** | **Responses (n)** |
| --- | --- |
| Research module/methods as part of training course | 82 |
| Research data protection/GDPR | 73 |
| Good Clinical Practice | 63 |
| Statistics | 26 |
| Survey/focus group training | 22 |
| Research specific postgraduate course | 13 |
| Academic writing/literature review | 11 |
| Research integrity/ethics | 9 |
| Technical research training | 8 |
| Patient and public involvement in research | 2 |
