# Supplementary material for: A vision for an academic health science centre: A survey of research engagement and barriers
Source: PLoS One. 2026 May 8;21(5):e0347753. doi: 10.1371/journal.pone.0347753 (PMC13155618; doi:10.1371/journal.pone.0347753)
Supplement: S3 Table — (DOCX) [file pone.0347753.s008.docx]

**S3 Table:** Barriers to research engagement

| **Barrier Identified** | **Responses (n) out of total of 20 identified barriers** | **Proportion (%) of total responses to this question** |
| --- | --- | --- |
| Time constraints | 47 | 30 |
| Lack of research support (including from management) | 18 | 12 |
| Not interested currently | 13 | 8 |
| Not enough resources | 12 | 8 |
| Institutional and ethical approval processes | 12 | 8 |
| Lack of knowledge/skills | 9 | 6 |
| General lack of opportunity | 7 | 4 |
| Lack of connectivity/joined up approach | 6 | 4 |
| Recent legislation | 5 | 3 |
| Research resources disproportionately distributed across the hospital | 5 | 3 |
| Not part of job | 5 | 3 |
| Lack of clinical academic pathways | 4 | 3 |
| Not inclusive enough | 3 | 2 |
| Better communication of research required | 3 | 2 |
| Paperwork/applications to begin projects | 2 | 1 |
| Others | 5 | 3 |
