# Supplementary material for: A vision for an academic health science centre: A survey of research engagement and barriers
Source: PLoS One. 2026 May 8;21(5):e0347753. doi: 10.1371/journal.pone.0347753 (PMC13155618; doi:10.1371/journal.pone.0347753)
Supplement: S4 Table — (DOCX) [file pone.0347753.s010.docx]

S4 Table: Participant suggestions for strategic research priorities

| **Suggested Strategy** | **Illustrative Quotation** |
| --- | --- |
| Increased institutional support for research | *"'Focus on communicating to all staff that research is not additional but part of their role. Need more supports and signposting for staff to engage in research."* |
| Increasing and maximising research resources and funding (incl from academic partners) | *"Research needs to be integrated into each clinical area and department with local champions working to improve and enhance awareness and use cases. There are many opportunities being lost due to lack of resources and this should be addressed"* |
| Better communication of ongoing research and research outcomes to patients and staff | *"I think an information desk on research in the concourse for both patients and staff would be useful. I know there are various targeted things from time to time. But maybe a research week where a few different disciplines can showcase their activities. An in-house conference of sorts? Mini lectures, presentations etc."* |
| Protected and more efficient use of research time for staff | *"Protected time as part of clinical contracts"* |
| Supporting a culture of research and innovation | *"There is massive data collected in departments, mainly on various excel sheets that is rarely used to inform, improve or update policies and practice due to a culture of 'that's how it is always been done', or 'no time', 'what's the use?'. A cultural shift is needed along with resources."* |
| Increased focus on interdisciplinary research and inclusivity including with our academic partner | *"Engaging all disciplines should be mandated to ensure inclusivity."* |
| Streamline research processes | *"Fast tracking assistance for low risk ethics and Data protection projects. A standard hospital wide policy on data protection for ethics board that is ‘how we do things’."* |
| Increased patient engagement and involvement and research | *"More media attention of research breakthroughs - to increase public interest in the hospital."* |
| Joint academic career pathways available to staff | *"The creation of joint clinical/academic posts or posts with protected research time across disciplines."* |
| Expansion of research topics/areas | *Trials should be conducted to maximise patient benefit and enrolment - they should be relative to the patients who actually attend SJH. Complex trials are often instigated for their prestige but enrol less than a handful of patients* |
| Staff acquisition and retention | *"We need to be cognisant of the "competition" from other sites across the state and actively pursue recruitment of world class clinician researchers"* |
| IT and data accessibility | *"Where possible clinical system databases need to be accessible to key users and resourced to provide relevant training on data extraction provided to ensure that rich data repositories are utilised for research and quality improvement"* |
| Importance of research translation | *"Ground roots, all very well research taking place but if it doesn't filter down to the wards and patients then no one benefits."* |
| Integration of research into clinical care processes | *"Making patient information and consent process easier using electronic systems and build biobanking into normal phlebotomy /sample process."* |
