# Supplementary material for: A vision for an academic health science centre: A survey of research engagement and barriers
Source: PLoS One. 2026 May 8;21(5):e0347753. doi: 10.1371/journal.pone.0347753 (PMC13155618; doi:10.1371/journal.pone.0347753)
Supplement: S6 Fig — (DOCX) [file pone.0347753.s012.docx]

**S6 Figure:** Participant feedback on Research Priority 4
